# Coexistence of Intestinal Spirochetosis and Colorectal Cancer: Could the Coil be Carcinogenic?

**DOI:** 10.14309/crj.0000000000001557

**Published:** 2024-11-16

**Authors:** Hannah Zuercher, Arvin Daneshmand, Eugene Stolow, Matthew Giansiracusa, Robert Allan, Antonios Sapounas

**Affiliations:** 1 Divison of Internal Medicine, University of Florida College of Medicine, Gainesville, FL; 2 Division of Gastroenterology, Hepatology, and Nutrition, University of Florida College of Medicine, North Florida/South Georgia Veterans Health System, Gainesville, FL; 3 Pathology and Laboratory Medicine Service, Department of Pathology, Immunology and Laboratory Medicine, University of Florida College of Medicine, North Florida/South Georgia Veterans Health System, Gainesville, FL

**Keywords:** intestinal spirochetosis, colorectal cancer, *Brachyspira*

## Abstract

Intestinal spirochetosis (IS) is an infectious gastrointestinal disease caused by *Brachyspira* bacteria. We detail an exceedingly rare case of IS with concomitant invasive colorectal adenocarcinoma (CRC) in a 58-year-old man presenting with abdominal discomfort and fever. Colonoscopic evaluation revealed abnormal-appearing, nodular cecal mucosa and a 35 mm rectosigmoid mass. Histopathology confirmed IS infection and CRC. Our case report is the first to detail IS diagnosed concurrently with colorectal cancer. It highlights the necessity of a high index of suspicion for IS in patients presenting with abdominal discomfort and endoscopic evidence of irregular nodular mucosa, particularly in the setting of suspected CRC. It further details potential pathophysiologic links between IS and colorectal malignancy.

## INTRODUCTION

Intestinal spirochetosis (IS) is caused by the enteric bacteria *Brachyspira aalborgior* and *Brachyspira pilosicoli.*^1^ Globally, the prevalence rate of IS ranges from 1% to 5% in developed areas.^2^ It remains debated whether this species is a pathogenic or commensal organism. Uncertainties regarding its pathogenicity stem from evidence of coinfection with other pathogens, including *Helicobacter pylori, Shigella flexneri,* and *Enterobius vermicularis*.^3,4^ The mechanism for IS pathogenicity consists of defacement of the brush border microvilli.^5^ Its transmission is currently poorly understood, though it is believed to be through the fecal-oral route.^6,7^ Notably, men who have sex with men and those with human immunodeficiency virus have higher prevalence rates, up to 62.5%.^6^

IS often presents clinically with progressive abdominal pain, watery diarrhea, and unintentional weight loss.^3^ The severity of symptoms fluctuates from asymptomatic to life-threatening manifestations.^8^ Bacteremia and end-organ failure have been detailed in case reports.^5,9^ IS can affect any portion along the proximal-to-distal colon, as well as the appendix.^10^ Present literature does not clearly define the relationship between IS and colorectal malignancy. However, evidence suggests that IS may correlate with enhanced colonic polyp development; hence, the importance of promptly identifying and treating IS.^11^

## CASE REPORT

**Figure 1.**
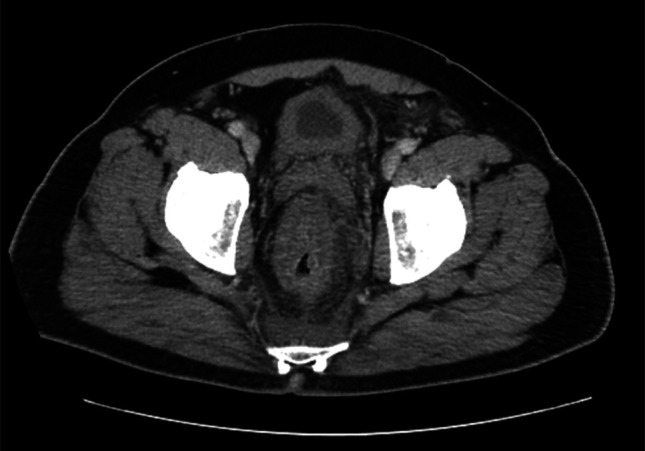
Computed tomography scan demonstrating marked wall thickening of the rectum and distal sigmoid colon, concerning for proctitis/colitis. There is a prominent amount of pericolonic and perirectal fat stranding also present, concerning for malignancy.

A 58-year-old man with a pertinent medical history of hypertension, prediabetes, and nondysplastic Barrett's esophagus (BE) presented with 8 days of cramping abdominal discomfort, subjective fevers up to 102°F, chills, myalgias, generalized weakness, and an increased stool frequency of 4 loose, nonbloody bowel movements daily. He reported a 60 pack-year smoking history and occasional alcohol use. The patient also reported high-risk sexual practices, including unprotected receptive anal intercourse with multiple male partners. A colonoscopy 4 years ago with excellent bowel preparation quality demonstrated 3 hyperplastic polyps each in the descending and sigmoid colons, and 3 in the rectum. Laboratory workup demonstrated a normal white blood cell count of 7,300 cells/mm³ (normal range is 4,500 to 11,000 cells/mm^3^) with a differential that was unremarkable for abnormalities. Blood cultures, urine studies, stool studies, and a respiratory viral panel were unremarkable. Recent hepatitis A, B, and C; HIV; chlamydia; gonorrhea; and syphilis testing was negative. Abdominopelvic computed tomography demonstrated marked wall thickening of the rectum and distal sigmoid colon concerning for proctitis/colitis, and an enlarged left internal iliac lymph node raising concern for an underlying rectosigmoid malignancy (Figure 1).

**Figure 2.**
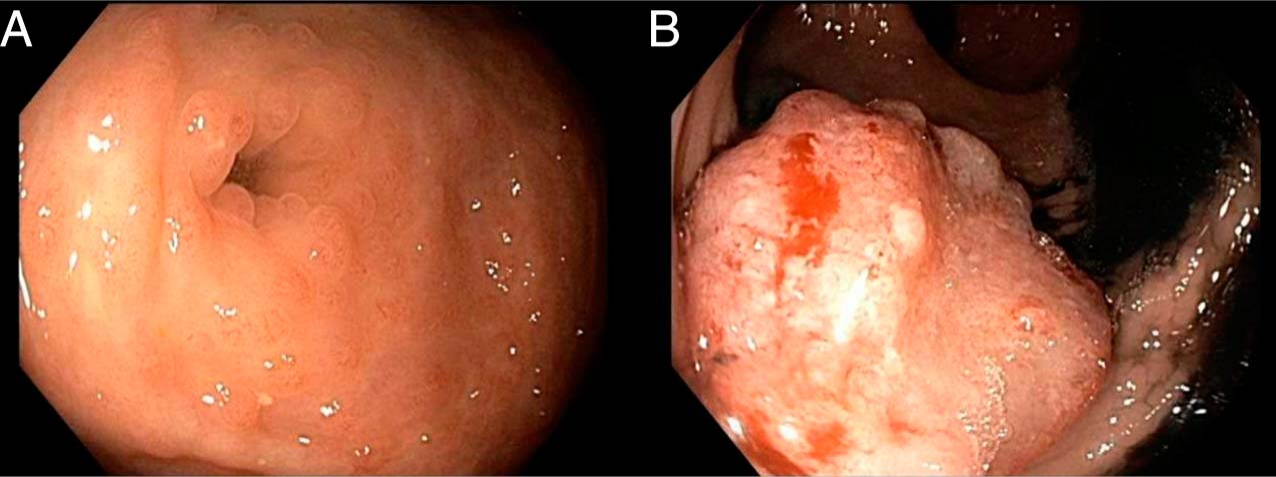
Colonoscopy demonstrating nodular cecal mucosa (part A) and a 35 mm rectosigmoid mass (part B).

**Figure 3.**
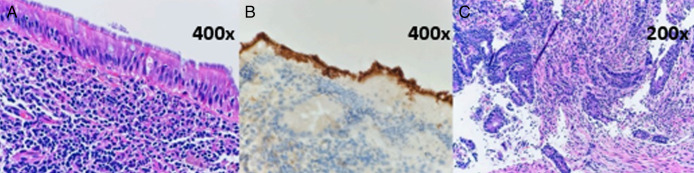
Hematoxylin and eosin stain showing mucosal lining with a shaggy appearance (part A, 400× magnification). Immunohistochemical stain for spirochetes demonstrating positive staining in the dense plaque of organisms (part B, 400× magnification). Invasive adenocarcinoma (part C, 200× magnification).

Esophagogastroduodenoscopy (EGD) and colonoscopy were subsequently performed (EGD for known BE-screening purposes). Although the EGD redemonstrated BE, the colonoscopy demonstrated a 15 mm cecal sessile lesion, mildly nodular cecal mucosa, sigmoid diverticulosis, and a 35 mm rectosigmoid mass (Figure 2). Biopsy of the sessile cecal mucosa demonstrated a sessile serrated lesion with dysplasia, while the nodular mucosa demonstrated IS with mildly active inflammation and reactive-appearing lymphoid aggregates (Figure 3). Biopsy of the rectosigmoid mass demonstrated moderately differentiated invasive adenocarcinoma involving the submucosa (Figure 3). After an infectious disease consultation, the patient was treated with a 10-day course of metronidazole 500 mg 3 times per day. Though treatment is not always required, it was recommended given underlying histopathological inflammation and potential immunosuppression associated with treatment for his colorectal cancer, which could foment disease progression. After appropriate staging (Stage IIIB, T3N1bM0), the patient underwent a sigmoidectomy with ileostomy placement. He tolerated this procedure well without any notable complications. Although the patient was initially hesitant to begin chemotherapy, he subsequently began adjuvant chemotherapy with folinic acid, fluorouracil, and oxaliplatin and reported overall clinical improvement. The patient noted that metronidazole also significantly improved his bowel movement quality and symptoms. Surveillance colonoscopy 6 months after colonic resection is pending.

## DISCUSSION

We report a case of a 58-year-old man with acute abdominal discomfort, watery diarrhea, and subjective fevers. The differential diagnosis in this clinical situation is broad, particularly given his significant social history. It is important to rule out the underlying infection before proceeding to endoscopic evaluation. Endoscopic cecal biopsies demonstrated IS. Given the patient's history of men who have sex with men, he was at a higher risk than the general population for symptomatic IS infection.^1,8,12^ Endoscopically, IS can appear grossly normal; however, mucosa has also been described as erythematous or polypoid.^10^ Diagnosis is achieved histologically using colonic tissue biopsies, with hematoxylin-eosin staining showing a diffuse blue fringe (a false brush border) along the intestinal cryptal-epithelial layer, as demonstrated in Figure 3.^1,3,13^ Subsequent stains for spirochetes are performed to confirm this diagnosis.^10,13^

Present literature is limited regarding the linkage between IS and malignancy. One recent study reported 3 cases of IS in patients with active or resolved malignancy, though none were colorectal malignancies.^1^ Another study supports the link between IS and sessile serrated adenomas/polyps, reporting a significantly higher rate of IS in such samples.^8,14,15^ The authors suggest that IS may be involved in the malignant alteration of such polyps.^5^ They posit, then, that eradication of IS holds great promise in decreasing overall risk of progression to colorectal adenocarcinoma (CRC). Last, one case study details a patient with IS treated with metronidazole, who was later diagnosed with mucinous adenocarcinoma. This was not previously seen on colonoscopy when the patient was infected with IS, suggesting that IS may be a causal factor in CRC, rather than a resulting one.^15^

Our case report is the first to detail IS diagnosed at the same time as CRC. Given the growing body of literature suggesting a link between IS and CRC, larger case studies may be beneficial to help elucidate this relationship and expand on clinical implications. It is believed that the inflammatory properties of IS increase one's risk of developing CRC, particularly if there is already polypoid tissue present. Given that the patient had a high-quality colonoscopy performed 4 years ago without a malignant lesion identified, it is plausible that IS could have accelerated and caused the development of advanced colon cancer in this short time period. Treatment of IS entails a course of antibiotics, typically a 10-day regimen of metronidazole.^3,16^ Notably, some patients achieve complete symptomatic improvement with antibiotic treatment, while others remain clinically symptomatic and with continued histologic demonstration of the false brush border.^16^ Although there are currently no definitive guidelines on the treatment of refractory IS, reports have detailed extended courses of up to 4 weeks of metronidazole or the addition of an amoxicillin regimen.^7^

In conclusion, this case highlights the importance of maintaining a broad differential diagnosis, including IS, when a patient with possible CRC presents with abdominal discomfort. IS formally diagnosed with endoscopic biopsy, and treatment with antibiotics can result in rapid improvement of symptoms. Evidence supporting the correlation between IS and CRC is limited but growing and warrants a larger scale study to explore this important relationship.

## DISCLOSURES

Author contributions: All authors listed on this manuscript have contributed significantly to the final product. Dr H. Zuercher is the article guarantor. H. Zuercher: abstract and manuscript writing, updating the reference list, and manuscript submission. A. Daneshmand: analyzing the patient's case in the electronic medical record system, finalizing the list of references, manuscript writing, editing and review. E. Stolow: manuscript editing and review. M. Giansiracusa: manuscript writing, editing and review. R. Allan: providing pathological images, manuscript editing, and review. A. Sapounas: performed the patient's colonoscopy and provided guidance, feedback, and review of the final submitted product.

Financial disclosure: None to report.

Informed consent was obtained for this case report.
